# Effects of Hostility and Lifestyle on Coronary Heart Disease among Middle-aged Urban Japanese

**DOI:** 10.2188/jea.11.243

**Published:** 2007-11-30

**Authors:** Nobuo Nishi, Shinsuke Nanto, Satoshi Shimai, Yumiko Matsushima, Keiko Otake, Akihito Ando, Katsuyuki Yamasaki, Sachiko Soga, Kozo Tatara

**Affiliations:** 1 Takarazuka City Health Promotion Center.; 2 Cardiovascular Division, Kansai Rosai Hospital.; 3 School of Human Sciences, Kobe College.; 4 Department of Human Relations, School of Letters, Mukogawa Women’s University.; 5 Department of Human Development, Naruto University of Education.; 6 Department of Psychology, Hyogo College of Medicine.; 7 Department of Social and Environmental Medicine, Osaka University Graduate School of Medicine.

**Keywords:** hostility, lifestyle, coronary heart disease, case-control study, Japanese

## Abstract

Objective: To explore the association between multi-dimensional aspects of hostility and coronary heart disease among middle-aged urban Japanese. Subjects and Methods: We conducted a case-control study. Cases were consecutive patients with acute coronary syndrome admitted to a hospital in Japan. Fifty-three patients (45 men and 8 women) aged 35 to 65 were enrolled. For each case, two sex and age (±2 years) matched controls were recruited from among participants in a health check-up program at a health promotion center located in the same area as the hospital. Two questionnaires, both with four components, were used to measure hostility and coping with anger: the one was for anger, hostility, physical aggression and verbal aggression, and the other for aggression, social inhibition, guilt, and controlled affect. Results: The scores of all components from two questionnaires were higher for cases than controls, but the differences were not significant. Multivariate analysis showed that anger, fat intake, alcohol consumption and house size were significantly associated with the etiology of acute coronary syndrome. Conclusion: Anger, lifestyle, and socioeconomic status play important roles for the etiology of coronary heart disease in middle-aged urban Japanese.

## INTRODUCTION

Hostility, a specific component of Type A behavior pattern, has been extensively studied for its possible adverse effect on coronary heart disease^1^^-^^5^^)^. A meta-analytic review by Miller et al.^6^^)^ showed that studies with different designs and measurement criteria for hostility and outcome have resulted in disparate findings. A recent systematic review by Hemingway and Marmot^7^^)^ reported a possible etiological role for (type A or) hostility in prospective cohort studies of health populations. So far, however, few studies from Japan have been published or included in a systematic review of this topic.

There seem to be two reasons for Japan falling behind in this field of research. The one is a relatively low coronary heart disease mortality rate in Japan^8^^)^, and the other a lack of appropriate instruments to measure hostility. Recently we have developed two types of questionnaires on hostility and completed an assessment of their validity and reproducibility: Japanese versions of the Buss-Perry Aggression Questionnaire (BAQ)^9^^, ^^10^^)^ and the Müller Anger Coping Questionnaire (MAQ)^11^^, ^^12^^)^. BAQ is a 24-item questionnaire and measures four components of aggression: anger, hostility, physical aggression, and verbal aggression. MAQ is a 23-item questionnaire and measures four components of coping with or expression of anger: aggression, controlled affect, guilt, and social inhibition

In this study we measured multi-dimensional aspects of hostility with the aid of these questionnaires and examined the effects of hostility and lifestyle, which has been reported as being closely associated with hostility^13^^-^^19^^)^, on the etiology of coronary heart disease.

## SUBJECTS AND METHODS

This study was based on data obtained from a hospital and a health promotion center, both of which are located in the same secondary medical care area of Japan. There are altogether 345 secondary medical care areas in Japan, and the study area, known as the Hanshin area, consists of seven cities and one town. The hospital is located in Amagasaki (pop. 488,586 in 1995) and functions as one of the tertiary care facilities for coronary heart disease in this area. The health promotion center is located in Takarazuka (pop. 202,544 in 1995), and provides health check-ups and exercise programs for local residents and workers.

### Cases

The cases consisted of consecutive patients with first acute coronary syndrome^20^^)^ admitted to the hospital between October 1997 and March 1999. Their diagnosis was based on 1) chest pain, 2) electrocardiographic findings, and 3) creatine phosphokinase. Our inclusion criteria were as follows: patients who 1) suffered from first acute coronary syndrome, 2) were aged 35 to 65 years, and 3) survived acute phase of the disease. From 63 eligible patients, 10 (16%) were excluded for refusal to the questionnaire or missing data. Then 53 patients (84%), 45 men and 8 women, were left for the analysis.

### Controls

Controls were selected from 1,011 individuals participating in a health check-up program at the health promotion center between September 1997 and March 1998. Two controls were randomly matched to each case for sex and age (±2 years) after exclusion of those who suffered from coronary heart disease.

### Questionnaire

We used the Japanese version^10^^)^ of the Buss-Perry Aggression Questionnaire (BAQ)^9^^)^ to investigate links between personality factors and health outcomes. It is a 24-item questionnaire and measures four components of aggression: anger, hostility, physical aggression, and verbal aggression. Anger is related to emotional aspects, hostility to cognitive aspects, and physical aggression and verbal aggression to behavioral aspects of hostility.

We also used the Japanese version^12^^)^ of the Müller Anger Coping Questionnaire (MAQ)^11^^, ^^21^^)^ to measure coping with or expression of anger. It is a 23-item questionnaire and measures four components: aggression, controlled affect, guilt, and social inhibition.

Information about lifestyle was obtained with a self-administered questionnaire. Smoking was categorized as 1) non-smoker, 2) ex-smoker, and 3) current smoker. Alcohol consumption was categorized as 1) non-drinker, 2) ex-drinker, 3) occasional drinker, and 4) almost daily drinker. Food intake was categorized as 1) not enough to feel satisfied, 2) moderate, and 3) almost always too much. Fat intake was categorized as 1) seldom, 2) sometimes, and 3) often. Regular exercise was categorized as 1) none, 2) less than once a week, 3) once a week, 4) twice a week, and 5) three times a week or more. House size was categorized as 1) less than 6 *tatamis* (one *tatami* is about 1.65 square meters), 2) 6 to 12 *tatamis*, 3) 13 to 18 *tatamis*, 4) 19 to 24 *tatamis*, 5) 25 to 30 *tatamis*, and 6) 31 *tatamis* and more.

The questionnaires were administered after the patients had been transferred from the coronary care unit to the general ward and before guidance was given about lifestyle or stress management in preparation for discharge.

### Statistical analysis

Statistical analysis was performed with SAS (SAS Institute, Cary, NC). Conditional logistic regression analysis^22^^)^ was performed using PROC PHREG. Variables on lifestyle and house size were dichotomized as followings: cigarette smoking (0= non- or ex-smoker, and 1= current smoker), alcohol consumption (0= non-, ex-, or occasional drinker, and 1= almost daily drinker), food intake (0= not enough to feel satisfied, or moderate, and 1= almost always too much), fat intake (0= seldom or sometimes, and 1= often), regular exercise (0= once a week or more, and 1= less than once a week), and house size (0= 25 *tatamis* or more, and 1= 24 *tatamis* or less).

## RESULTS

BAQ and MAQ scores for both cases and controls are shown in Table 1. The scores of all components were higher for cases than controls, but the differences were not significant.

**Table 1.** The Buss-Perry Aggression Questionnaire (BAQ) and the Müller Anger Coping Questionnaire (MAQ) scores in a case-control study for the etiology of coronary heart disease among middle-aged urban Japanese.

	Cases (n=53)		Controls (n=106)		P value*
	
	Mean	SD	Mean	SD	
BAQ					
Anger	15.1	4.0	14.0	3.7	0.087
Hostility	15.9	2.8	15.8	3.3	0.861
Physical aggression	16.4	4.6	15.5	3.5	0.194
Verbal aggression	16.5	3.0	15.7	2.9	0.158
MAQ					
Aggression	6.8	3.5	6.1	3.5	0.240
Social inhibition	9.4	3.0	8.6	3.1	0.114
Guilt	9.2	3.2	8.9	3.3	0.573
Controlled affect	4.8	2.0	4.2	1.9	0.058

*Student’s *t*-test

Among the items related to lifestyle or socioeconomic status, cigarette smoking, fat intake, regular exercise, and house size were the factors with significant differences between cases and controls (Table 2). Current smokers were more prevalent in cases than in controls. The percentage of persons who ate fatty foods often was higher in cases, although the percentage of those who did so seldom was also higher in this group. The percentage of those who exercise once a week or more was higher for controls (37.7%) than for cases (32.1%), as was the percentage of those living in a house with 25 *tatamis* or more (69.8% for controls and 45.3% for cases).

**Table 2.** Lifestyle and socioeconomic status in a case-control study for the etiology of coronary heart disease among middle-aged urban Japanese.

	Cases (n=53)		Controls (n=106)		P value*
	
	N	%	N	%	
Cigarette smoking					
Non-smoker	15	28.3	32	30.2	0.041
Ex-smoker	3	5.7	21	19.8	
Current smoker	35	66.0	53	50.0	
Alcohol intake					
Non-drinker	24	45.3	36	34.0	0.106
Ex-drinker	1	1.9	1	0.9	
Occasional	16	30.2	24	22.6	
Almost daily	12	22.6	45	42.5	
Food intake					
Not enough to feel satisfied	19	35.8	50	47.2	0.075
Moderate	18	34.0	40	37.7	
Almost always too much	16	30.2	16	15.1	
Fat intake					
Seldom	16	30.2	20	18.9	0.000
Sometimes	18	34.0	73	68.9	
Often	19	35.8	13	12.3	
Regular exercise					
No exercise	29	54.7	62	58.5	0.037
Less than once a week	7	13.2	4	3.8	
Once a week	5	9.4	12	11.3	
Twice a week	2	3.8	16	15.1	
Three times a week or more	10	18.9	12	11.3	
House size (number of *tatamis*)					
Less than 6	1	1.9	0	0.0	0.000
6 - 12	10	18.9	2	1.9	
13 - 18	9	17.0	8	7.5	
19 - 24	9	17.0	22	20.8	
25 - 30	7	13.2	20	18.9	
31 or more	17	32.1	54	50.9	

*Chi-square test

Finally, multivariate analysis by conditional logistic regression was performed. Among the components of BAQ and MAQ, anger in BAQ and controlled affect in MAQ were entered into the model because they were the factors showing marginally significant differences between cases and controls (Table 1). We created dichotomous variables by splitting the factors at the median: anger (0= less than 15 points, and 1= 15 points or more) and controlled affect (0= less than 5 points, and 1= 5 points or more). Application of the stepwise conditional logistic regression model resulted in anger, alcohol intake, fat intake, and house size remaining as significant risk factors (Table 3).

**Table 3.** Odds ratios (95% C.I.) by conditional logistic model in a case-control study for the etiology of coronary heart disease among middle-aged urban Japanese.

	Odds ratio (95% C.I.)
Anger	
Lower (score<=14)	1.00 (Reference)
Higher (score>=15)	2.33 (1.06 - 5.12)
Alcohol intake	
Non-, ex-, and occasional drinker	1.00
Almost daily drinker	0.32 (0.12 - 0.84)
Fat intake	
Seldom or sometimes	1.00
Often	4.12 (1.52 - 11.14)
House size	
25 *tatamis* or more	1.00
24 *tatamis* or less	2.96 (1.38 - 6.36)

The following dichotomous variables were entered but removed from the model: controlled affect (0 = less than 5 points, and 1=5 points or more), cigarette smoking (0 = non - or ex-smoker, and 1 = current smoker), food intake (0 = not enough to be satisfied, or moderate, and 1 = almost always too much), regular exercise (0 = once a week or more, and 1 = less than once a week).

## DISCUSSION

The effects of hostility and lifestyle on the etiology of coronary heart disease in middle-aged urban Japanese were examined in a case-control study. Although the Ho scale by Cook and Medley^23^^)^ has been widely used to measure hostility, we used BAQ and MAQ to measure multi-dimensional aspects of hostility. Among the components of BAQ and MAQ, multivariate analysis identified anger as a significant variable. This is in concordance with previous data of cohort design^4^^, ^^24^^)^.

Among lifestyle factors, multivariate analysis showed that alcohol consumption had a strong palliative effect (OR=0.32), although the effect was not estimated by separating ex-drinkers from non- or occasional drinkers. Moderate alcohol drinking has been demonstrated to prevent coronary heart disease^25^^-^^27^^)^. Among urban Japanese middle-aged men, which are similar to our sample, Kitamura et al. has reported that alcohol drinking reduces a risk of coronary heart disease^27^^)^ although a statistical significance was found only for moderate drinkers. Our findings seem to agree with their results. Saturated fat intake has been reported to be a risk factor for coronary heart disease^28^^, ^^29^^)^. Our results also showed fat intake to be an important predictor of coronary heart disease (OR=4.12).

Although smoking is a major risk factor for coronary heart disease and Japan is notorious for its high prevalence in men (a little lower than 60%), it did not turn out to be significant. One reason might be due to a strong association between cigarette smoking and other variables such as alcohol drinking, house size, and hostility^17^^)^. Another reason might be that our multivariate model did not have a statistical power to detect cigarette smoking as a risk factor due to a small number of subjects.

There have been few studies on socioeconomic gradients of coronary heart disease in Japan. In our study house size, measured in number of *tatamis*, was used as an indicator of socioeconomic status, and was identified by multivariate analysis as a significant factor. Amagasaki and Takarazuka belong to the same secondary medical care area, but Amagasaki contains an industrial area along Osaka Bay, while Takarazuka is mainly a residential area. According to the statistics, average number of rooms in a household was 3.63 in Amagasaki and 4.56 in Takarazuka in 1995^30^^)^. Even though our results must have been somewhat affected by this difference between the two cities, it is unlikely that the odds ratio of 2.96 can be explained only by this difference. This study could be the first of a series to determine whether socioeconomic status can affect the etiology of coronary heart disease among Japanese.

A major limitation of this study is a retrospective nature of data collection for cases. The patients could estimate their hostility either higher or lower after their onset of acute coronary syndrome. As the scores in BAQ and MAQ were supposed to be, indeed they were, higher in cases than in controls (Table 1), we took some measures to avoid the estimation of higher score. First, we asked the patients to answer their behaviors not after the onset but at about half a year before. Second, we delivered the questionnaires not at an acute phase in a coronary care unit but at a stable phase in a general ward. Third, we gave an instruction on lifestyles or stress management after the questionnaires were completed.

The small number of cases can also be a limitation of our study, as described above. This is not only because of the relatively low mortality from coronary heart disease in Japan but also because of the age range. We aimed to study the effects of hostility on rather younger patients, so we restricted cases to an age range of 35 to 65, as done by Meesters and Smulders^31^^)^. This restriction also resulted in excluding many female patients who were relatively older than male patients. So we could not analyze the data for men and for women separately. In addition, we could have conducted a multi-center study to increase the number of cases. But we restricted our study to one hospital with a cooperative staff because careful instructions were essential for the patients to answer the questionnaire accurately. As the role of sex on hostility could be important, this limitation should be solved in studies with more samples in the future.

In conclusion, anger, lifestyle, and socioeconomic status play important roles in the etiology of coronary heart disease among middle-aged urban Japanese. Further studies are necessary, however, to explore in more detail the development of coronary heart disease among Japanese.
